# Comparison of outcomes between transcatheter tricuspid valve repair and surgical tricuspid valve replacement or repair in patients with tricuspid insufficiency

**DOI:** 10.1186/s13019-023-02271-9

**Published:** 2023-04-29

**Authors:** Xiqiang Wang, Yanpeng Ma, Zhongwei Liu, Xiude Fan, Gongchang Guan, Shuo Pan, Junkui Wang, Yong Zhang

**Affiliations:** 1 Department of Cardiovascular Medicine, Shaanxi Provincial People’s Hospital, Xi’an, Shaanxi People’s Republic of China; 2 Department of Endocrinology, Shandong Provincial Hospital Affiliated to Shandong First Medical University, Jinan, Shandong People’s Republic of China

**Keywords:** Transcatheter tricuspid valve repair, Surgical tricuspid valve replacement, Surgical tricuspid valve repair, Tricuspid insufficiency

## Abstract

**Background:**

Tricuspid regurgitation is associated with significant morbidity and mortality, but with limited treatment options. The objective of this study is to compare the demographic characteristics, complications, and outcomes of transcatheter tricuspid valve repair (TTVr) versus surgical tricuspid valve replacement (STVR) or surgical tricuspid valve repair (STVr), using real-world data from the National Inpatient Sample (NIS) database.

**Methods and results:**

Our study analyzed data from the National Inpatient Sample (NIS) database from 2016 to 2018 and identified 92, 86, and 84 patients with tricuspid insufficiency who underwent STVr, STVR, and TTVr, respectively. The mean ages of patients who received STVr, STVR, and TTVr were 65.03 years, 66.3 years, and 71.09 years, respectively, with TTVr patients significantly older than those who received STVr (*P* < 0.05). Patients who received STVr or STVR had higher mortality rates (8.7% and 3.5%, respectively) compared to those who received TTVr (1.2%). Patients who underwent STVr or STVR were also more likely to experience perioperative complications, including third-degree atrioventricular block (8.7% STVr vs. 1.2% TTVr, *P* = 0.329; 38.4% STVR vs. 1.2% TTVr, *P* < 0.05), respiratory failure (5.4% STVr vs. 1.2% TTVr, *P* = 0.369; 15.1% STVR vs. 1.2% TTVr, *P* < 0.05), respiratory complications (6.5% STVr vs. 1.2% TTVr, *P* = 0.372; 19.8% STVR vs. 1.2% TTVr, *P* < 0.05), acute kidney injury (40.2% STVr vs. 27.4% TTVr, *P* = 0.367; 34.9% STVR vs. 27.4% TTVr, *P* = 0.617), and fluid and electrolyte disorders (44.6% STVr vs. 22.6% TTVr, *P* = 0.1332; 50% STVR vs. 22.6% TTVr, *P* < 0.05). In addition, the average cost of care and the average length of hospital stay were higher for patients who underwent STVr or STVR than for those who received TTVr (USD$37995 ± 356008.523 STVr vs. USD$198397 ± 188943.082 TTVr, *P* < 0.05; USD$470948 ± 614177.568 STVR vs. USD$198397 ± 188943.082 TTVr, *P* < 0.05; 15.4 ± 15.19 STVr vs. 9.6 ± 10.21 days TTVr, *P* = 0.267; 24.7 ± 28.81 STVR vs. 9.6 ± 10.21 days TTVr, *P* < 0.05).

**Conclusion:**

TTVr has shown to have favorable outcomes compared to STVr or STVR, but more research and clinical trials are required to help formulate evidence-based guidelines for the role of catheter-based management in tricuspid valve disease.

**Supplementary Information:**

The online version contains supplementary material available at 10.1186/s13019-023-02271-9.

## Introduction

Moderate or severe tricuspid regurgitation (TR) is observed in 0.55% of the general population and its prevalence increases with age, affecting about 4% of the patients aged 75 years or more [1], approximately 1.6 million people in the United States and 3 million people in Europe with clinically relevant tricuspid regurgitation [2, 3]. There is increasing evidence demonstrates that tricuspid regurgitation is not only a marker of concurrent cardiac disease, but also a potential driver of major adverse cardiovascular events [4, 5].

At present, few treatment options exist for tricuspid regurgitation, patients with mild or moderate TR are often treated conservatively with medical therapies [6]. Surgeries such as surgical tricuspid valve replacement (STVR) or surgical tricuspid valve repair (STVr) are considered more definitive treatment in patients with severe TR [7, 8]. However, evidence shows that tricuspid valve surgery with a peri-operative mortality rate of 8–10% [9, 10], and STVR is at risk for biological valvular degeneration, thrombosis, and long-term anticoagulation of mechanical valves, and the risk of reoperation increased in STVr [11, 12].

Therefore, minimally invasive catheter therapy could effectively reduce tricuspid regurgitation and lower the risk of perioperative complications. In recent years, various minimally invasive catheter techniques have been applied to reduce tricuspid regurgitation [3, 6, 13−16]. For example, annular reduction procedures had shown a promising in reducing tricuspid regurgitation and had clinical benefits for tricuspid regurgitation patients [17, 18]. Because leaflet malcoaptation is the main pathology of tricuspid regurgitation, the edge-edge clip technique, which is used to treat functional mitral regurgitation [3], has also been used to treat tricuspid regurgitation, and its results have been reported in retrospective studies [3, 19].

Although initially promising, most transcatheter tricuspid valve repair (TTVr) approaches are still in development and the outcomes and safety evaluations of TTVr versus STVR or STVr remain limited and lack of support from randomized controlled trials or other high-quality clinical studies. The aim of the present study is to evaluate the burden, outcomes, financial cost and complications of TTVr versus STVR or STVr in a real-world population from the National Inpatient Sample (NIS) database.

## Methods

### Study Data

In this study, we used the NIS data from January 2016 to December 2018 which was developed by the Agency from Healthcare Research and Quality of the United States through a federal-state-industry partnership. The NIS database has more than 8 million inpatients and represents 20% of all hospital admissions in the United States. And it is updated annually, so we can use these data to analyze the disease trend over time. Because the NIS database is publicly available, we do not need to get the approval of the institutional review board or the informed consent in our clinical study.

### Study design and data selection

**Fig. 1:**
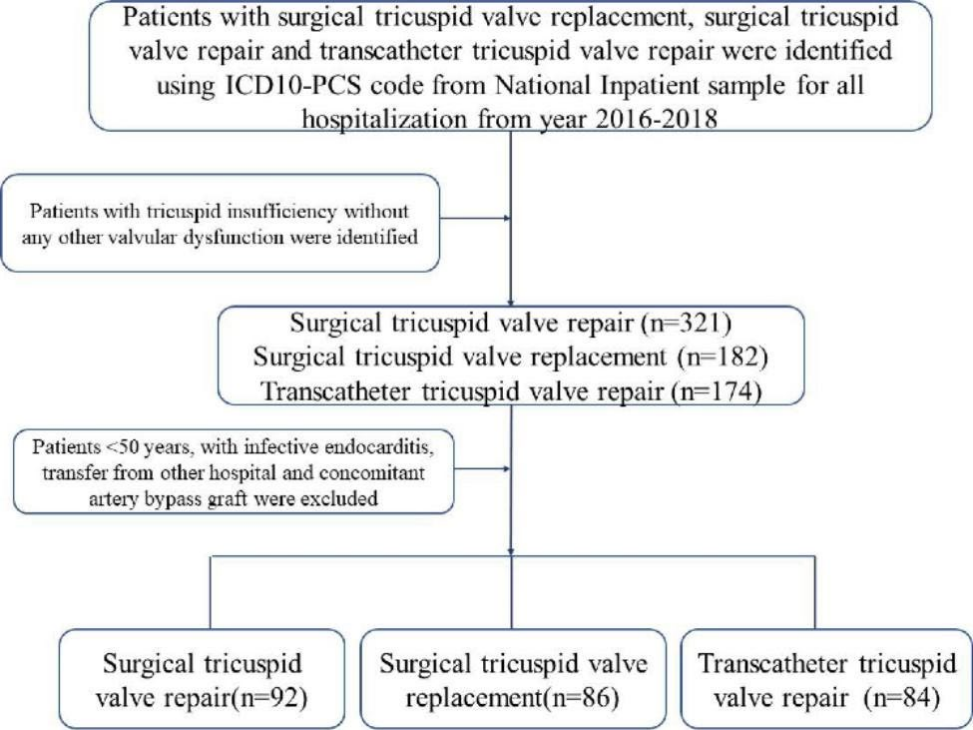
Flowchart of the study cohort ICD 10-PCS indicates International Classification of Diseases, Tenth Revision, Procedure Coding System

The International Classification of Diseases, Tenth Revision, Clinical Modification (ICD-10-CM) codes and ICD-10-Procedure Coding System (PCS) codes were used to analysis these data. The NIS data from 2016 to 2018 were used in the present study (Table S1). Patients with tricuspid valve insufficiency but without any other valvular disease were selected using ICD-10-CM code (I340, I051, I341, I342, I050, I351, I061, I350, I060, I352, I062, I361, I071, I360, I070, I362, I072). Patients who underwent TTVr, STVr, and STVR were selected by ICD-10-PCS codes (02QJ3ZG, 02QJ3ZZ, 02QJ3ZG), ICD-10-PCS codes (02QJ0ZG, 02QJ0ZZ), and ICD10-PCS codes (02RJ07Z, 02RJ0JZ), respectively. The patients who younger than 50 years old, with infective endocarditis, or with coronary artery bypass surgery previously, and/or other valvular diseases were excluded from our study. A flowchart of our patient selection criterion is presented in Fig. 1.

### Study outcomes

The primary endpoints of our study were in-hospital mortality and periprocedural complications. The secondary outcomes of interest were resource use and operative procedures related trends over time, such as length of hospital stay, total charges.

### Statistical analysis

The Kolmogorov-smirnov test was used to test whether the variables were normally distributed. Normal distribution variables were expressed as the mean ± standard deviation (SD), and Student’s T test was used for comparisons between groups. The baseline characteristics among the 3 groups were analyzed by the Kruskal-Wallis test for nonparametric variables, one-way ANOVA for parametric variables, and the χ [2] exact test was used for categorical variables. For all analyses, a 2-sided *P* value of 0.05 was considered statistically significant. Statistical analyses were performed using SPSS version 25 (IBM, Armonk, NY) and R version 3.5 (version 3.6.3, R Core Team).

## Results

### Characteristics of study participants selected from NIS database

Between January 2016 and December 2018, a total of 262 patients who underwent tricuspid valve procedures were identified (Fig. 1). Of these, 92 patients underwent STVr (35.1%), 86 (32.8%) patients had STVR procedures and there were 84 (32.1%) patients underwent TTVr surgery (Fig. 1; Table 1). Patients underwent TTVr were older compared to those who underwent STVr procedures (71.09 years vs. 66.3 years, *P* = 0.029), there was a trend to decrease in patients who had STVR surgery compared with the patients who underwent TTVr (66.3 years vs. 71.09 years, *P* = 0.128). Both cohorts included predominantly White patients (65.2% STVr vs. 61.6% STVR vs. 57.1% TTVr) (Table 1). Use of STVr, STVR, TTVr were similar among Hispanic patients (8.7% vs. 8.1% and 14.3, Table 1).

Among the STVr, STVR, TTVr groups, the patients were more diagnosed with coronary artery disease (43.5% STVr vs. 26.7% STVR vs. 42.9% TTVr), left heart failure (56.5% STVr vs. 58.1% STVR vs. 85.7% TTVr), hyperlipemia (45.7% STVr vs. 26.7% STVR vs. 64.3% TTVr), atrial fibrillation (57.6% STVr vs. 91.9% STVR vs. 78.6% TTVr) and renal failure (45.7% STVr vs. 50.0% STVR vs. 71.4% TTVr) (Table 1). Compared with STVr group, the TTVr group with more female (*P* < 0.05, Table 1), more diagnosed with left heart failure (*P* < 0.05, Table 1), and chronic obstructive pulmonary disease (*P* < 0.05, Table 1). However, compared with STVR group, the TTVr group with more diagnosed with hyperlipemia and less permanent pacemaker implantation (*P* < 0.05, Table 1).

**Table 1:** Basic Characteristics of the Patients Who Underwent STVr, STVR and TTVr (2016–2018)

Characteristic	STVr (n = 92)	STVR (n = 86)	TTVr (n = 84)	*P* Value
Age, yrs (mean ± SD)	65.03 ± 9.22*	66.30 ± 9.6	71.09 ± 10.68	0.086
Female sex, n (%)	40 (43.5) *	40 (46.5) *	66 (78.6)	0.049
**Race**				0.12
White	60 (65.2)	53 (61.6)	48 (57.1)	
African American	12 (13.0)	13 (15.1)	6 (7.1)	
Hispanic	8 (8.7)	7 (8.1)	12 (14.3)	
Asian/Pacific Islander	2 (2.2)	7 (8.1)	1 (1.2)	
Native American	0 (0)	1 (1.2)	6 (7.1)	
Other races	10 (10.9)	13 (15.1)	12 (14.3)	
**Comorbidities and medical history**				
Hypertension, n (%)	31 (33.7)	26 (30.2)	12 (14.3)	0.4
Diabetes mellitus, n (%)	27 (29.3)	20 (23.3)	24 (28.6)	0.826
Coronary artery disease, n (%)	40 (43.5)	23 (26.7)	36 (42.9)	0.334
Myocardial infarction, n (%)	5 (5.4)	7 (8.1)	1 (1.2)	0.691
Left heart failure, n (%)	52 (56.5) *	50 (58.1)	72 (85.7)	0.103
Hyperlipemia, n (%)	42 (45.7)	23 (26.7) *	54 (64.3)	0.608
Cerebral hemorrhage, n (%)	2 (2.2)	3 (3.5)	6 (7.1)	0.353
Cerebral infarction, n (%)	2 (2.2)	1 (1.2)	1 (1.2)	0.723
Atrial fibrillation, n (%)	53 (57.6)	79 (91.9)	66 (78.6)	0.001
Liver disease, n (%)	16 (17.4)	17 (19.8)	6 (7.1)	0.616
Renal failure, n (%)	42 (45.7)	43 (50.0)	60 (71.4)	0.199
Peripheral vascular disease, n (%)	2 (2.2)	1 (1.2)	1 (1.2)	0.926
Chronic obstructive pulmonary disease, n (%)	12 (13)*	17 (19.8)	30 (35.7)	0.9
Deficiency anemia, n (%)	7 (7.6)	7 (8.1)	1 (1.2)	0.746
Coagulopathy, n (%)	21 (22.8)	13 (15.1)	6 (7.1)	0.393
Obesity, n (%)	15 (16.3)	10 (11.6)	18 (21.4)	0.699
Alcohol use, %	1 (1.1)	1 (1.2)	1 (1.2)	1
Tobacco abuse, n (%)	26 (28.3)	13 (15.1)	24 (28.6)	0.417
Permanent pacemaker implantation	7 (7.6)	30 (34.9) *	6 (7.1)	0.001
ICD implantation	9 (9.8)	3 (3.5)	1 (1.2)	0.511
**Primary payer, n (%)**				0.96
Medicare	50 (54.3)	43 (50)	47 (56.0)	
Medicaid	7 (7.6)	7 (8.1)	6 (7.1)	
Private insurance	33 (35.9)	30 (34.9)	6 (7.1)	
Other	2 (22.2)	7 (8.1)	6 (7.1)	

STVr indicates surgical tricuspid valve repair; STVR indicates surgical tricuspid valve replacement; TTVr indicates transcatheter tricuspid valve repair. *P < 0.05, vs. TTVr.

### Clinical outcomes in Study Cohort

#### Trends in STVr, TTVr and STVR

**Fig. 2:**
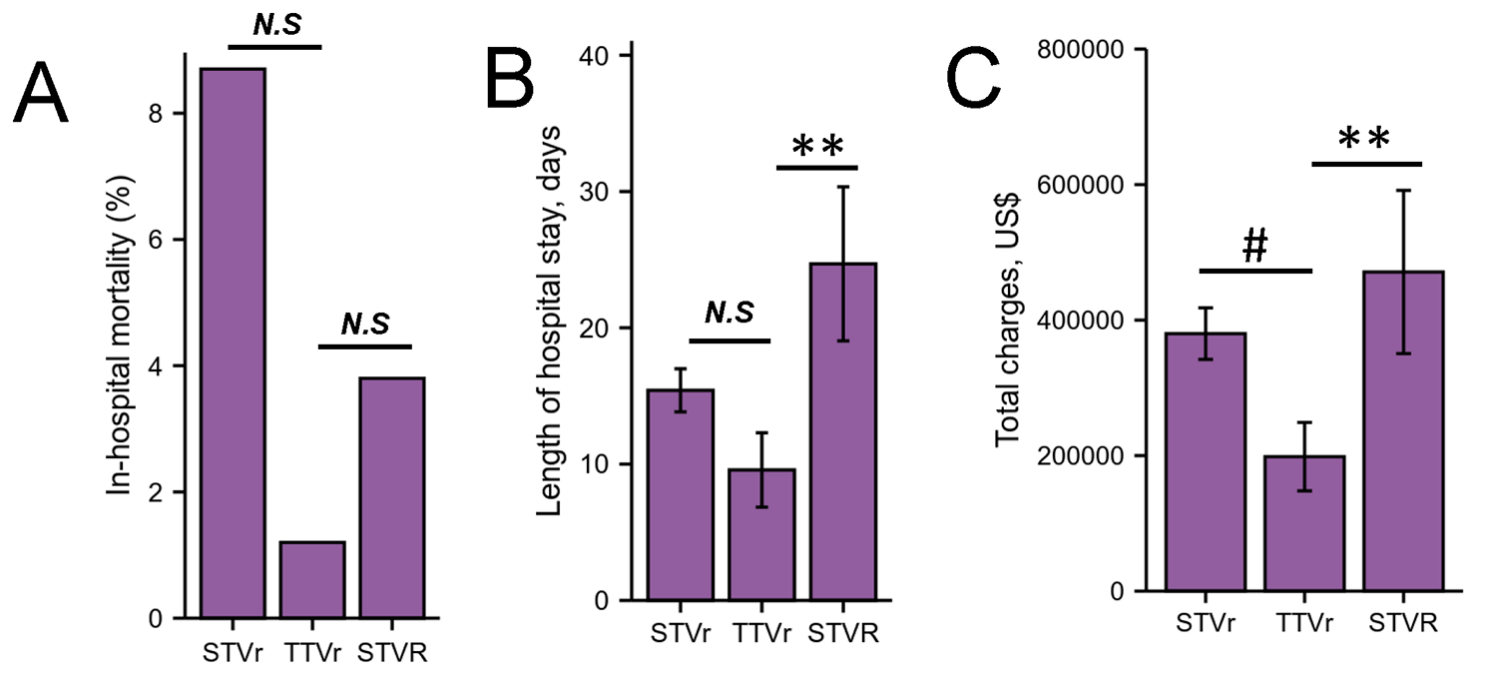
Trends in STVr, TTVr and STVR. A, In-hospital mortality in patients undergoing STVr, TTVr and STVR from 2016 to 2018. B, Length of stay in patients undergoing STVr, TTVr and STVR from 2016 to 2018. C, Trends in cost of stay in patients undergoing STVr, TTVr and STVR from 2016 to 2018. STVr indicates surgical tricuspid valve repair; STVR indicates surgical tricuspid valve replacement; TTVr indicates transcatheter tricuspid valve repair

There was no significant difference of in-hospital mortality in STVr, STVR and TTVr, however, the in-hospital mortality in TTVr had a decreasing trend when compared with STVr and STVR (Table 2; Fig. 2A). Length of stay (15.41 ± 15.193 days STVr vs. 9.57 ± 10.211 days TTVr, *P* = 0.267; 24.69 ± 28.807 days STVR vs. 9.57 ± 10.211 days TTVr, *P* < 0.05, Table 2; Fig. 2B) and cost of care ($379994.53 ± 365008.523 STVr vs. $198396.71 ± 188943.082 TTVr, *P* < 0.05; $470947.27 ± 614177.568 STVR vs. $198396.71 ± 188943.082 TTVr, *P* < 0.01, Table 2; Fig. 2C) were considerably higher for STVr, and STVR when compared with TTVr.

**Table 2:** Clinical Outcomes in Patients Who Underwent STVr, STVR and TTVr (2016–2018)

Variable	STVr (n = 92)	STVR (n = 86)	TTVr (n = 84)	*P* Value
In-hospital mortality, n (%)	8 (8.7)	3 (3.5)	1 (1.2)	0.32
Length of hospital stay, days	15.41 ± 15.193	24.69 ± 28.807*	9.57 ± 10.211	0.025
Total charges, US$	379994.53 ± 365008.523*	470947.27 ± 614177.568*	198396.71 ± 188943.082	0.142
Cardiac tamponade, n (%)	3 (3.3)	6 (7.0)	6 (7.1)	0.297
Cardiogenic shock, n (%)	18 (19.6)	13 (15.1)	12 (14.3)	0.938
Cardiac arrest, n (%)	2 (2.2)	3 (3.5)	6 (7.1)	0.353
Third degree atrioventricular block, n (%)	8 (8.7)	33 (38.4)*	1(1.2)	< 0.001
Respiratory failure, n (%)	5 (5.4)	13 (15.1)*	1 (1.2)	0.154
Respiratory complications, n (%)	6 (6.5)	17 (19.8)*	1 (1.2)	0.085
Mechanical ventilation use, n (%)	12 (13)	23 (26.7)	6 (7.1)	0.184
IABP, n (%)	8 (8.7)	3 (3.5)	6 (7.1)	0.972
ECMO, n (%)	4 (4.3)	1 (1.2)	2 (2.4)	0.73
Pericardial complications, n (%)	11 (12)	17 (19.8)	12 (14.3)	0.563
Hemopericardium, n (%)	1 (1.1)	1 (1.2)	1 (1.2)	0.936
Bleeding/hematoma post-procedure, n (%)	5 (5.4)	10 (11.6)	6 (7.1)	0.466
Thrombosis due to cardiac prosthetic devices, n (%)	1 (1.1)	1 (1.2)	1 (1.2)	0.936
Acute embolism and thrombosis, n (%)	2 (2.2)	1 (1.2)	6 (7.1)	0.374
Blood transfusion, n (%)	18 (19.6)	7 (8.1)	6 (7.1)	0.304
Acute kidney injury, n (%)	37 (40.2)	30 (34.9)	23 (27.4)	0.762
Fluid and electrolyte disorders, n (%)	41 (44.6)	44 (51.2)*	19 (22.6)	0.302

STVr indicates surgical tricuspid valve repair; STVR indicates surgical tricuspid valve replacement; TTVr indicates transcatheter tricuspid valve repair; ECMO, extracorporeal membrane oxygenation; IABP, intra-aortic balloon pump. *P < 0.05, vs. TTVr.

### Cardiac complications in STVr, TTVr and STVR

**Fig. 3:**
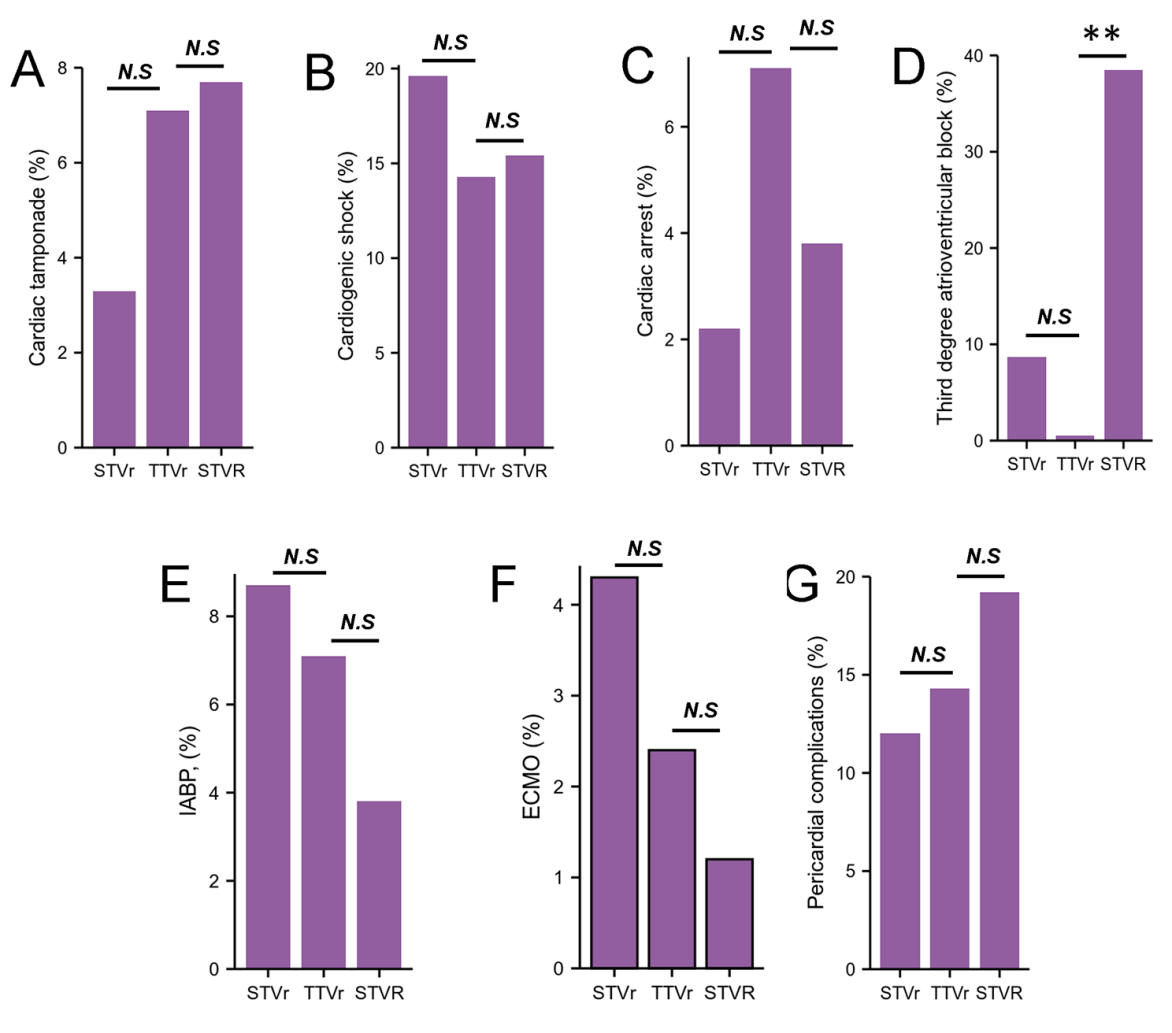
Cardiac complications in STVr, TTVr and STVR. A, Cardiac tamponade in patients undergoing STVr, TTVr and STVR from 2016 to 2018. B, Cardiogenic shock in patients undergoing STVr, TTVr and STVR from 2016 to 2018. C, Cardiac arrest in patients undergoing STVr, TTVr and STVR from 2016 to 2018. D, Third degree atrioventricular block in patients undergoing STVr, TTVr and STVR from 2016 to 2018. E, IABP implantation in patients undergoing STVr, TTVr and STVR from 2016 to 2018. F, ECMO implantation in patients undergoing STVr, TTVr and STVR from 2016 to 2018. G, Pericardial complication in patients undergoing STVr, TTVr and STVR from 2016 to 2018. STVr indicates surgical tricuspid valve repair; STVR indicates surgical tricuspid valve replacement; TTVr indicates transcatheter tricuspid valve repair

There was no significant difference for the cardiac tamponade, cardiogenic shock, cardiac arrect between STVr, STVR and TTVr (Table 2; Fig. 3A-C), however, the third-degree atrioventricular block in TTVr were considerably lower when compared with STVr (8.7% STVr vs. 1.2% TTVr, *P* = 0.329, Table 2; Fig. 3D) and STVR (38.4% STVR vs. 1.2% TTVr, *P* < 0.05, Table 2; Fig. 3D). Intra-aortic balloon pump (IABP) was required in 7.1% of patients who underwent TTVr, 8.7% of STVr and 3.5% of STVR (Table 2; Fig. 3E). Usage rate of extra corporeal membrane oxygenation (ECMO) was higher for STVr (4.3% STVr, 1.2% STVR, 1.2% TTVr, *P* = 0.73, Table 2; Fig. 3F), although there was no difference between these groups. There was no significant difference for the pericardial complication between STVr, STVR and TTVr (Table 2; Fig. 3G).

### Respiratory complications in STVr, TTVr and STVR

**Fig. 4:**
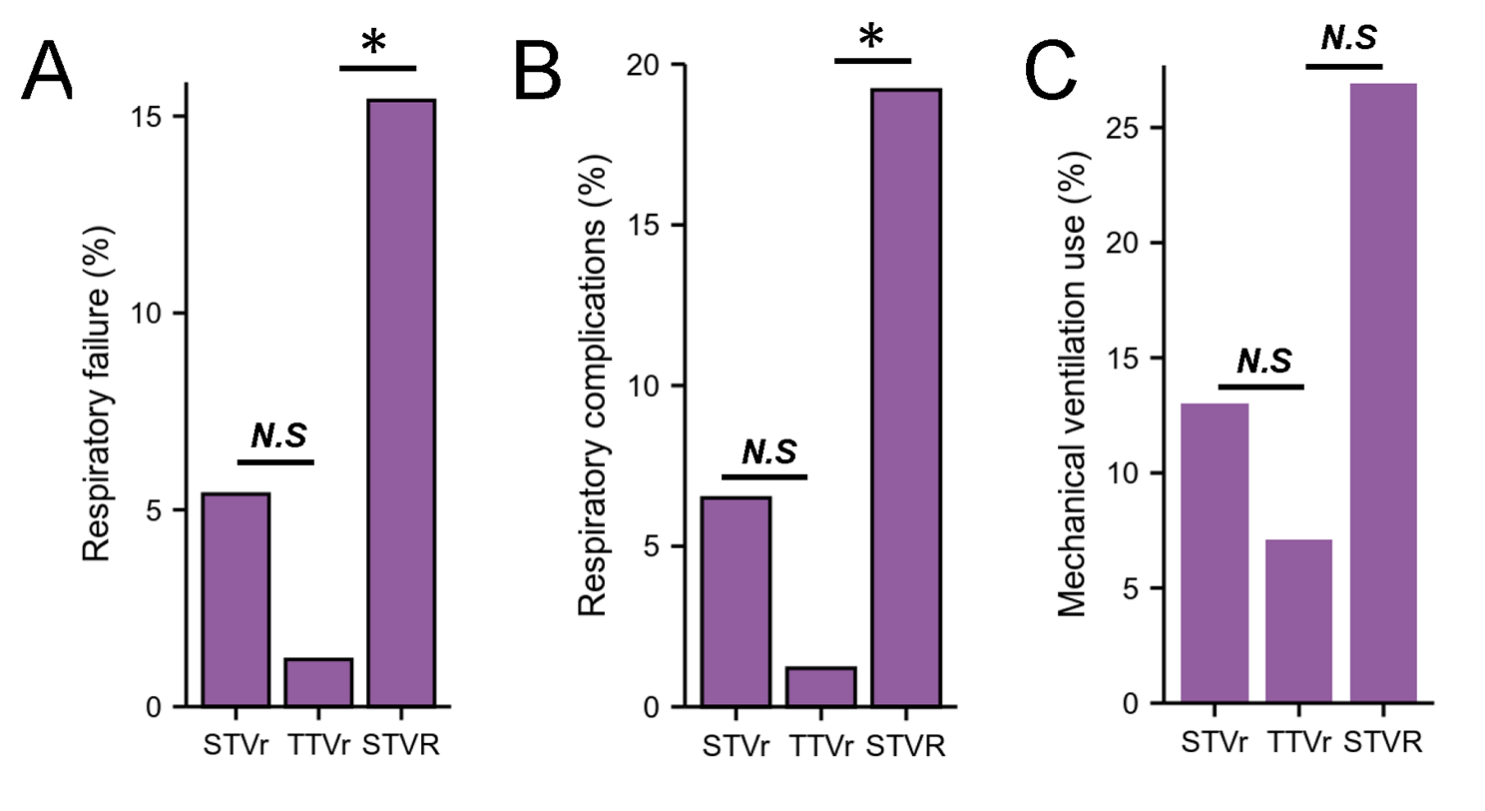
Respiratory complications and Device implantation in STVr, TTVr and STVR. A, Respiratory failure in patients undergoing STVr, TTVr and STVR from 2016 to 2018. B, Respiratory complications in patients undergoing STVr, TTVr and STVR from 2016 to 2018. C, Mechanical ventilation use in patients undergoing STVr, TTVr and STVR from 2016 to 2018. STVr indicates surgical tricuspid valve repair; STVR indicates surgical tricuspid valve replacement; TTVr indicates transcatheter tricuspid valve repair

The patients who underwent STVr and STVR were more likely to suffer from respiratory failure (5.4% STVr vs. 1.2% TTVr, *P* = 0.369; 15.1% STVR vs. 1.2% TTVr, *P* < 0.05, Table 2; Fig. 4A), respiratory complications (6.5% STVr vs. 1.2% TTVr, *P* = 0.372; 19.8% STVR vs. 1.2% TTVr, *P* < 0.05, Table 2; Fig. 4B), and mechanical ventilation use (13% STVr vs. 7.1% TTVr, *P* = 0.531; 26.7% STVR vs. 7.1% TTVr, *P* = 0.136, Table 2; Fig. 4C).

### Other perioperative complications in STVr, TTVr and STVR

**Fig. 5:**
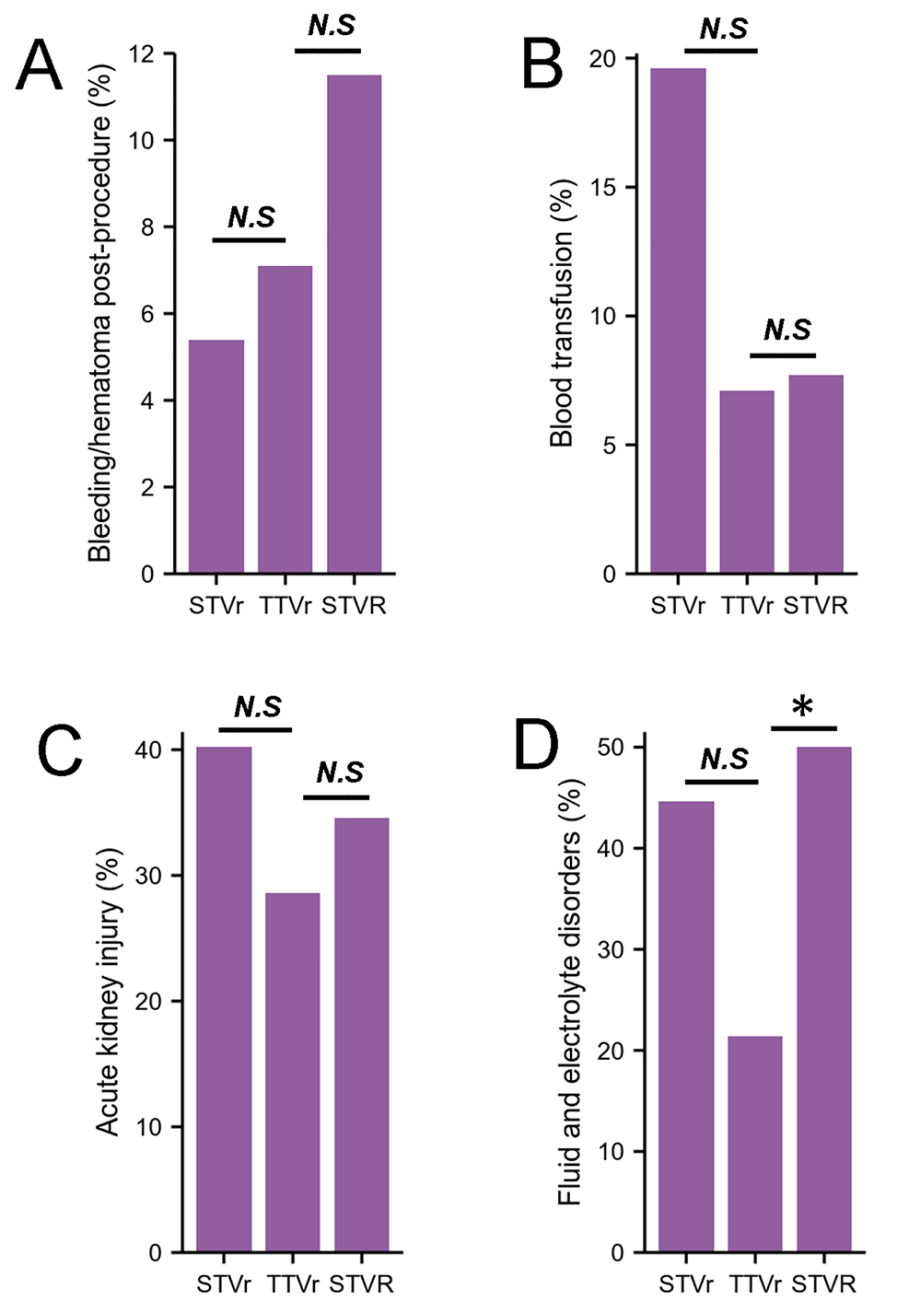
Other perioperative complications in STVr, TTVr and STVR. A, Bleeding/hematoma post-procedure in patients undergoing STVr, TTVr and STVR from 2016 to 2018. B, Blood transfusion in patients undergoing STVr, TTVr and STVR from 2016 to 2018. C, Acute kidney injury in patients undergoing STVr, TTVr and STVR from 2016 to 2018. D, Fluid and electrolyte disorders in patients undergoing STVr, TTVr and STVR from 2016 to 2018. STVr indicates surgical tricuspid valve repair; STVR indicates surgical tricuspid valve replacement; TTVr indicates transcatheter tricuspid valve repair; ECMO, extracorporeal membrane oxygenation; IABP, intra-aortic balloon pump

There was no significant difference for the bleeding/hematoma post-procedure, blood transfusion in STVr, STVR and TTVr (Table 2; Fig. 5A-B), but it seems that TTVr had the lower rate of acute kidney injury (40.2% STVr vs. 34.9% TTVr, *P* = 0.405; 34.9% STVR vs. 28.6% TTVr, *P* = 0.697, Table 2; Fig. 5C) and fluid and electrolyte disorders (44.6% STVr vs. 21.4% TTVr, *P* = 0.102; 50.0% STVR vs. 21.4% TTVr, *P* < 0.05, Table 2; Fig. 5D) when compared with STVr and STVR.

## Discussion

The following main findings were reported for the first time in our contemporary real-world study of outcomes for TTVr vs. STVr or STVR. (1) The in-hospital mortality was lower for patients who underwent TTVr when compared with STVr or STVR. (2) TTVr was related with lower periprocedural complications. (3) The length of stay in hospital and medical cost were significantly higher for STVr or STVR compared to TTVr.

Given the high risk of tricuspid surgery [20] and poor outcomes with conservative therapy [21], transcatheter tricuspid intervention has recently emerged as a viable alternative. At present, transcatheter leaflet repair was proved to be the most common strategy for some of the tricuspid insufficiency patients, with excellent safety and site-reported procedural success (TR grade ≤ 2^+^) ranging from 72–86% [22]. However, most TTVr approaches are still in development and the outcomes and safety evaluations of TTVr versus STVR or STVr remain limited and lack of support from randomized controlled trials or other high-quality clinical studies. Using the NIS database, the present study elaborates the outcomes and use of resources of TTVr compared with STVr or STVR. In a nationally representative sample of US hospitalizations, the total charge and the length of hospital days of TTVr were significantly lower than STVR, compared with STVr, the total charge and the length of hospital days of TTVr seems to decreasing, but not with significantly difference, representing the better potential adoption of TTVr at the national level in the US.

Similar to other reports [23, 24], compared with STVR or STVr, our study demonstrated that patients who underwent TTVr were older and had higher burden of comorbidities such as heart failure, hyperlipemia, renal failure, and chronic obstructive pulmonary disease suggesting that a larger proportion of patients may be at higher risk of surgery. Although patients who underwent TTVr were older than those who underwent STVR or STVr groups, the TTVr group has higher in-hospital mortality, indicating that STVR or STVr maybe far behind TTVr in surgical safety, but more clinical studies were needed to confirm the result. At present, there was no study to compare the mortality of TTVr versus surgical tricuspid valve procedure, one study demonstrated that isolated tricuspid valve surgery being a rare procedure with an in-hospital mortality of nearly 10% [25], but TTVr seems to offer symptomatic improvement and a reduction in heart failure related hospitalizations with a low rate of complications and mortality [15], these results were similar with our data.

Third degree atrioventricular block and respiratory complications maybe the scariest perioperative complications of tricuspid valve surgical or procedures, but these complications impact on early mortality has not been well investigated. Our results demonstrated that the early third-degree atrioventricular block rate for current STVR and STVr recipients were approximately 38.5% and 8.7%, which was higher than that in TTVr series. STVR and STVr also had higher respiratory complications (STVR of 19.2, STVr of 6.5%) when compared with TTVr. Further clinical studies are needed to confirm above-mentioned perioperative complications.

The preliminary studies with TTVr demonstrated that despite the high-risk profile of TR patients undergoing TTVr, most procedures were well tolerated and associated with lower in-hospital mortality [26]. To date, the International Multi-site transcatheter Tricuspid Valve Registry (NCT03416166) represents the largest cohort of patients treated with TTVr using different devices aomng the world [27]. The last report of the study (n = 470) demonstrated that the included patients exhibited a high estimated surgical risk, and 73% of them had been admitted for right ventricle failure before the procedure [28]. The results displayed that the procedural success was obtained in 80% of the included patients, and the TriClip system was used in 79% of the patients. In-hospital and 30-day mortality was 3.2% and 3.8%, respectively. Also, several factors were identified as the predictors of follow-up survival [28]: the presence of ascites (HR = 3.10; 95% CI, 1.50–16.50; *P* = 0.01), baseline systolic pulmonary artery pressure (HR = 16.20; 95% CI, 2.00-135.80; *P* = 0.01), procedural success (HR = 0.22; 95% CI, 0.01–4.50; *P* < 0.01).

Although the feasibility and initial efficacy of TTVr have been well documented, data on clinical outcomes with extended follow-up are still scarce. Orban et al. [29] showed the effect of transcatheter edge-to-edge repair on the rate of heart failure hospitalization in 119 patients. And among them 93% patients with TriClip system and 7% patients with Pascal system. The study demonstrated that the average annual hospitalization rate of heart failure was decreased by 22%, from 1.21 to 0.95 per patient per year (*P* = 0.02). Also, TR grade reduction persisted at 1-year follow-up (72% with moderate or less TR grade) and NYHA class improved significantly (grade I-II in 67% of patients at 1 year vs. 9% at baseline; *P* < 0.001). In addition, 72% of the patients persisted with moderate or less TR grade at 1-year follow-up and 67% of the patients with grade I-II of NYHA class which was improved significantly compared with baseline (*P* < 0.001).

In the absence of RCT studies, Taramasso et al. compared the outcomes of TTVr with conservative treatment got from 2 large medical centres [30]. As a results, a total of 268 patients were identified from the 2 medical centres, the data shown that compared with control patients, TTVr patients had lower 1-year mortality (23 ± 3% vs. 36 ± 3%; *P* = 0.001) and rehospitalization (26 ± 3% vs. 47 ± 3%; *P* < 0.0001)^30^. These data indicated that TTVr associated with lower mortality and heart failure rehospitalization compared with medical therapy, which should be confirmed in future RCT studies.

There are some limitations of our study because of the inherent weakness of NIS database and the study design. The major limitations of our study include small sample size, observational design, and a lack of standardized protocols for patient management. In addition, there is no data for laboratory and echocardiography in this study to compare the cardiac function among the groups. Furthermore, the long-term endpoints could not be evaluated in NIS samples because NIS database was not designed to follow up patients’ data longitudinally and we didn’t have the information about surgery via conventional sternotomy or minimally invasive methods.

## Conclusion

TTVr has shown to have favorable outcomes compared to STVr or STVR, but more research and clinical trials are required to help formulate evidence-based guidelines for the role of catheter-based management in tricuspid valve disease.

## Electronic supplementary material

Below is the link to the electronic supplementary material.

Additional File 1: Criteria to determine presence of diseases in NIS database.

## Data Availability

All data generated or analyzed during this study are included in this published article.
